# Dietary *Bacillus subtilis* PB6 Enhances Reproductive Performance by Modulating Gut Microbiota, Barrier Function, and Inflammation in *Clostridium perfringens Type A*-Infected Sows

**DOI:** 10.3390/ani16071032

**Published:** 2026-03-27

**Authors:** Mengran Zhang, Aohang Yu, Chihao Wang, Chaojie Chen, Chenchen Wu

**Affiliations:** College of Animal Veterinary Medicine, Northwest A&F University, Yangling 712100, China

**Keywords:** sow, *Clostridium perfringens type A*, *Bacillus subtilis*, PB6, piglet

## Abstract

*Bacillus subtilis* is aerobic or facultatively anaerobic. After entering the gastrointestinal tract, its spores germinate and colonize the gut, inhibiting the growth of harmful aerobic bacteria (*Escherichia coli*, *Streptococcus*, *Staphylococcus aureus*). However, it remains unclear whether *B. subtilis* can inhibit *Clostridium perfringens type A* infection. In this study, *B. subtilis* PB6 was added to the diets of pregnant sows infected with *Clostridium perfringens type A*, which significantly improved the reproductive performance and reduced the incidence of bloat in sows and diarrhea in neonatal piglets. The treatment significantly increased the abundance of intestinal probiotics (*B. subtilis*, *Lactobacillus*, *Limosilactobacillus reuteri*, *Lactobacillus johnsonii*, *Muribaculaceae*, *Lactobacillus amylovorus*, and *Lactobacillus reuteri*) in sows and decreased the relative abundance of *Clostridium perfringens type A* after feeding *B. subtilis* administration. These probiotics can repair the intestinal tissue and improve intestinal histomorphology, and enhance the expression of MUC2 and sIgA in sows, thereby further strengthening the mucosal immune function. *B. subtilis* can also reduce the levels of inflammatory factors (CRP, IL-1β, and IFN-γ) and attenuate the inflammatory response in sows and neonatal piglets. Taken together, our results suggest that dietary supplementation with *B. subtilis* PB6 could reduce bloat in sows and diarrhea in piglets while improving intestinal barrier function and microbial balance in sows.

## Simple Summary

The *Bacillus subtilis* treatment significantly increased the abundance of intestinal probiotics in sows and decreased the relative abundance of *Clostridium perfringens type A* after feeding *B. subtilis*. The reduction in *Clostridium perfringens type A* abundance led to a significant decrease in the α-toxin levels. These probiotics can improve the intestinal tissue and intestinal histomorphology and enhance the expression of MUC2 and sIgA in sows, further strengthening the intestinal mucosal immune function. *B. subtilis* can also attenuate the levels of inflammatory factors and the inflammatory response in sows and neonatal piglets. Taken together, our results suggest that dietary supplementation with *B. subtilis* PB6 could reduce bloat in sows and diarrhea in piglets while improving intestinal barrier function and microbial balance in sows.

## 1. Introduction

*Clostridium perfringens type A* is one of the main pathogenic bacteria causing diarrhea in piglets. The incidence is highest, especially in neonatal piglets within the first three days of life [1,2,3]. *Clostridium perfringens type A* infection in piglets leads to high mortality, causing significant economic losses to the swine industry. Morbidity can reach 100%, while mortality rates can exceed 70% and even approach 100% [4]. *Clostridium perfringens type A* is widely found in the intestinal lumen of animals and humans, as well as in the natural environment, and is a conditionally pathogenic bacterium.

Traditional methods for preventing diarrhea in piglets involve supplementing diets with antibiotics, but long-term or excessive use can lead to bacterial resistance and antibiotic residues in animal products [5]. Hence, identifying safe and effective alternatives for preventing diarrhea in weaned piglets is urgent. *B. subtilis* is aerobic or facultatively anaerobic. After entering the gastrointestinal tract, its spores colonize the gut, consuming large amounts of free oxygen and inhibiting the growth of harmful aerobic bacteria (*Escherichia coli*, *Streptococcus*, *Staphylococcus aureus*). This creates a favorable environment for other beneficial bacteria (*Lactobacillus*, *Bifidobacterium*), which may explain the observed reduction in diarrhea incidence in young livestock and poultry [6]. However, it remains unclear whether *B. subtilis* can inhibit the growth of *Clostridium perfringens type A.* Therefore, dietary supplementation with PB6 was evaluated for its effects on growth performance, gut health, and gut functions in neonatal piglets by modulating the gut microbiota and its metabolic activity. We aimed to investigate the effects of *B. subtilis* PB6 on the bloating rate, reproductive performance, growth performance, intestinal histomorphology, and fecal microbial composition in sows and their piglets.

## 2. Materials and Methods

### 2.1. Experimental Design

The *B. subtilis* PB6 strain used in this study was isolated from the intestines of healthy animals. Initial experiments were conducted on a sow farm where 60 fecal samples were randomly collected from sows at different reproductive stages: early gestation (0–30 days), mid-gestation (31–90 days) and late gestation (91–114 days), and lactation. A *Clostridium perfringens* was detected using a rapid detection kit.

Subsequently, 40 pregnant sows (parity 2–3, body weight 200–220 kg) were randomly assigned to either a control group (n = 20 sows) and an experimental group (*B. subtilis* group sows, n = 20 sows). The experimental group received PB6 via feed mixing at 0.6 g/kg feed for one month, while the control group was fed a standard late-gestation diet. Both the *B. subtilis* and the control groups were challenged with *Clostridium perfringens type A*. The farrowing performance of sows and the growth performance of piglets were monitored. The farrowing house was equipped with semi-slotted mesh flooring and maintained at a temperature of 20–24 °C and humidity of 40–60%. Each farrowing crate measured 2 m × 2 m, and sows had free access to feed and water. Routine farm management practices for treatment and immunization were followed. The vaccination schedule for sows was implemented as follows:

Four weeks before breeding: Inactivated porcine rotavirus vaccine (targeting G4, G5, and G9 strains) was administered at a dose of 2 mL via intramuscular injection in the neck (manufactured by Keqian, Wuhan, China).

Three weeks before breeding: Inactivated trivalent vaccine (against *Escherichia coli*, *Clostridium perfringens type C*, and *Clostridium novyi*) was administered at a dose of 2 mL via intramuscular injection in the neck (manufactured by Hipra, Ames, IA, USA).

During the delivery process, there were staff specifically responsible for assisting with delivery, such as injecting oxytocin and providing manual farrowing assistance. If the placenta was not discharged during sow delivery and the interval between the delivery of two piglets was more than 3 h, artificial assistance was provided. The delivery personnel worked in three shifts to ensure that there were staff members supervising the delivery process at all times. Written informed consent was obtained from the owners for the participation of their animals in this study.

### 2.2. Ethics Statement

The studies involving animals were reviewed and approved by the Committee on Laboratory Animal Welfare and Ethics of the Chinese Society for Laboratory Animals and Northwest A&F University (approval no.abc2024-7-18). Euthanasia methods for piglets followed scientific humane principles to ensure a painless death. Cardiac arrest was triggered by an overdose of intravenous sodium pentobarbital (>150 mg/kg) or by high concentrations of potassium chloride (2 mmol/kg) administered intravenously after anesthesia. During the operation, the piglets immediately lost consciousness and stopped breathing.

### 2.3. Ethics and Consent to Participate

All test sows were owned by the farm owner, and official consent documents signed by the owner had been obtained. The purpose of this experiment is to apply *B. subtilis* PB6 for better prevention of production practices such as bloating and diarrhea in sows caused by *Clostridium perfringens A*. All experimental sows complied with animal welfare standards to minimize stress and ensure feeding conditions as much as possible, and fully complied with the production standard of the farm.

### 2.4. Production Performance of Sows

Production performance data were collected at three time points: 1 month before PB6 feeding initiation (baseline), and after one and two months of continuous PB6 supplementation. The flatulence mortality rates, number of live-born piglets, birth weight, daily diarrhea, mortality of piglets, diarrhea rate and death rate were calculated. Weaned piglets were weighed to determine average daily gain (ADG).

The flatulence mortality rate = (number of sows dying from bloat/total number of dead sows) × 100%. We determined whether the cause of death in pigs was bloating through the following aspects: abdominal distension with tension, percussion sound of “drum sound” at the site of bloating, no depression or rapid recovery of depression after pressing, and synchronous stiffness and bloating.

The neonatal birth weight = total of individual weights of all piglets within 24 h of birth; the diarrhea rate = total number of days with diarrhea/(number of experimental pigs × number of experimental days) × 100%; the number of days with diarrhea refers to one piglet having diarrhea per day, which is one head day.

The death rate = (total number of dead piglets/total number of piglets) × 100%; the ADG (g/d) = total weight gain/number of trial days.

### 2.5. Sample Collection

Blood samples (5 mL) were collected via anterior vena cava puncture from both the *B. subtilis* group and control group pregnant sows (5 sows per group), as well as their 7-day-old and 20-day-old neonatal piglets (5 piglets per age group per maternal group, yielding a total of 30 samples). Samples were kept at 4 °C overnight, centrifuged at 3000 r/min for 20 min, and the supernatant (serum) was aliquoted into cryotubes and stored at −20 °C for later analysis.

Intestines (ileum, colon), liver, heart, and spleen were collected from bloat dead and healthy sows in two groups during necropsy. Tissues were preserved in fixative solutions. Part of the intestinal samples were used for histopathological test via HE staining, part for mucous layer and goblet cell analysis via PAS staining, and part for secretory immunoglobulin A (sIgA) distribution and expression detection via immunohistochemistry (IHC).

### 2.6. Analysis of Clostridium perfringens Type A Levels

A total of 60 fresh fecal samples from pregnant sows (30 from early pregnancy and 30 from mid-late pregnancy) were collected and analyzed using the Clostridium perfringens rapid detection kit from Jianming Technology Co., Ltd. (Zhuhai, China). The detection principle of this kit relies on the specific metabolic characteristic of *C. perfringens* (i.e., decomposition of sulfur-containing compounds to produce hydrogen sulfide, which reacts with ferrous ions in reagents to form black ferrous sulfide precipitates). The entire procedure (including sample homogenization, reaction condition control, and quality control setup) was performed in accordance with the classic method for *C. perfringens* detection described in ISO 15213-2:2023 (Table 1) standard [7,8]. For judging the results, the presence and intensity of black precipitates in reaction tubes were used as core criteria: positive control tubes (inoculated with *C. perfringens* standard strain ATCC 13124 (CRL-2936^TM^, Xi’an, China, 2024), consistent with the kit’s built-in quality control system) were required to show typical black precipitates, while negative control tubes remained colorless.

### 2.7. Intestinal Microbiota Analysis

To comprehensively analyze the effects of *Clostridium perfringens type A* intervention on the structure and function of intestinal microbiota in sows and piglets, the 16S rRNA sequencing was used for deep profiling of fecal microbial communities. The specific procedures were as follows:

#### 2.7.1. Sample Pretreatment and DNA Extraction

(1) Collect fresh fecal samples from different litters of piglets on the 7th day after delivery and the day before weaning (3 samples per group), and from sows on the day before weaning (3 samples per group). Fecal samples were collected from 3 independent sows/groups, while piglet samples were collected from 3 different litters/groups to ensure biological reproducibility. Samples were immediately placed into sterile cryotubes, snap-frozen in liquid nitrogen, and stored at −80 °C.

(2) DNA extraction: Microbial total DNA was extracted using the TIANamp Stool DNA Kit (DP328, TIANGEN Biotech, Beijing, China) according to the manufacturer’s instructions: 200 mg fecal sample was added to 1.5 mL lysis buffer (Containing proteinase K), vortexed for 5 min to mix thoroughly; lysed at 65 °C for 30 min with vortexing every 10 min; 200 μL buffer GB was added, vortexed, incubated at 70 °C for 10 min, and centrifuged (12,000× *g*, 4 °C, 5 min) to collect the supernatant; DNA was purified via an adsorption column and eluted with 50 μL Buffer TE, and then stored at −20 °C.

(3) DNA quality detection: concentration and purity were assessed.

#### 2.7.2. Library Construction and Sequencing

DNA fragmentation and library preparation: DNA was randomly fragmented into 350 bp fragments using a Covaris M220 sonicator (Covaris, Woburn, MA, USA). After end repair, A-tailing, and Illumina adapter ligation (San Diego, CA, USA), target fragments (300–400 bp) were selected using AMPure XP magnetic beads (Beckman Coulter, Brea, CA, USA). PCR amplification (8–10 cycles) was performed with KAPA HiFi HotStart ReadyMix (Roche, Basel, Switzerland) to construct paired-end (PE) sequencing libraries. Libraries were pooled, denatured, and sequenced on an Illumina NovaSeq 6000 platform (Illumina, San Diego, CA, USA) with a target data volume of ≥10 Gb/sample (performed by Shanghai Majorbio Bio-Pharm Technology Co., Ltd., Shanghai, China). Data analysis was conducted using QIIME 2, and species annotation was based on the Greengenes database (version 13.8). The Shannon diversity index and Simpson index were calculated.

#### 2.7.3. qPCR

Total RNA was extracted from sow feces using Trizol reagent (Takara, Maebashi, Japan) according to the manufacturer’s protocol. First-strand cDNA was synthesized from total RNA using a cDNA synthesis kit (Bio-Rad, Hercules, CA, USA) according to the manufacturer’s instructions. The UltraSYBR mixture was used for PCR amplification of 2 µL cDNA in a 25 µL reaction volume. The PCR mixture was denatured at 94 °C for 2 min, followed by 40 cycles of 94 °C for 30 s, 60 °C for 30 s, and 72 °C for 45 s. The relative quantification of bacterial 16S rRNA genes was performed using the 2^−ΔΔCT^ method. The average CT values from three technical replicates were used for each biological replicate. The primers used are listed in Table 2. Primer specificity was verified by NCBI Primer BLAST, and annealing temperature was optimized by gradient PCR to 60 °C.

### 2.8. Analysis of Inflammatory and Nonspecific Immune Factors

Serum samples from control and experimental pregnant sows and neonatal piglets were collected via anterior vena cava puncture. Levels of immunoglobulin G (IgG), immunoglobulin M (IgM), C-reactive protein (CRP), procalcitonin (PCT), interleukin-6 (IL-6), serum amyloid A (SAA), tumor necrosis factor-α (TNF-α), interferon-γ (IFN-γ), interleukin-1β (IL-1β), interleukin-2 (IL-2), interleukin-8 (IL-8) and cortisol were measured using ELISA kits.

ELISA Assay: The concentration of target analyte (such as IL-6, PCT, CRP) in serum samples was quantified using the Jingmei II ELISA Kit (Jiangsu Jingmei Biotechnology Co., Ltd., Yangzhou, China; Cat. No. JM-EL-IL6-II). All operations (including sample dilution, incubation time, and color development) strictly followed the manufacturer’s official manual. The absorbance was measured at 450 nm using a microplate reader (Thermo Fisher Scientific, Waltham, MA, USA), and the concentration was calculated based on the standard curve.

### 2.9. Histomorphometry

After necropsy, intestinal tissues from piglets were immersed in 10% neutral buffered formalin for fixation. Tissue blocks were prepared via trimming, dehydration, clearing, wax infiltration, and embedding. HE staining was performed to observe changes in intestinal villi and tissue structure.

HE Staining: Intestinal tissue samples were fixed in 10% neutral buffered formalin for 24 h at room temperature. After routine paraffin embedding, 4 μm-thick sections were prepared using a microtome. HE staining was performed following the standard protocol described by Su et al. [2] (2020, Veterinary Pathology). Images were captured using an optical microscope at 200× magnification.

### 2.10. Immunohistochemical (IHC) Analysis

Intestinal tissues from sows were fixed in 10% neutral buffered formalin, processed into paraffin-embedded blocks, and sectioned (4 μm). IHC staining was performed to detect the distribution and expression of MUC2 and sIgA proteins, following standard protocols with minor modifications. Briefly, sections underwent deparaffinization, rehydration, antigen retrieval, non-specific binding blocking, incubation with primary and horseradish peroxidase (HRP)-conjugated secondary antibodies, 3,3′-diaminobenzidine (DAB) color development, and hematoxylin counterstaining. Images were captured under an optical microscope at 200× and 400× magnifications. The optical density (OD) of positively stained areas was quantified using ImageJ software (version 1.54), and the expression levels of MUC2 and sIgA were represented as the average OD value.

### 2.11. Statistical Analysis

The normality test of the data was conducted using the Shapiro–Wilk test, the Student’s *t*-test was used for comparison between two groups, and the One Way ANOVA+Tukey’s post hoc test was used for multi-group comparison, with *p* < 0.05 indicating a significant difference. • *p* > 0.05: No significant difference between the experimental and control groups at the same time point; • *p* < 0.05: statistically significant difference between the experimental and control groups at the same time point.

## 3. Results

### 3.1. Effects of PB6 on Reproductive Performance of Sows Infected with Clostridium perfringens Type A and Growth Performance of Their Piglets

**Figure 1:**
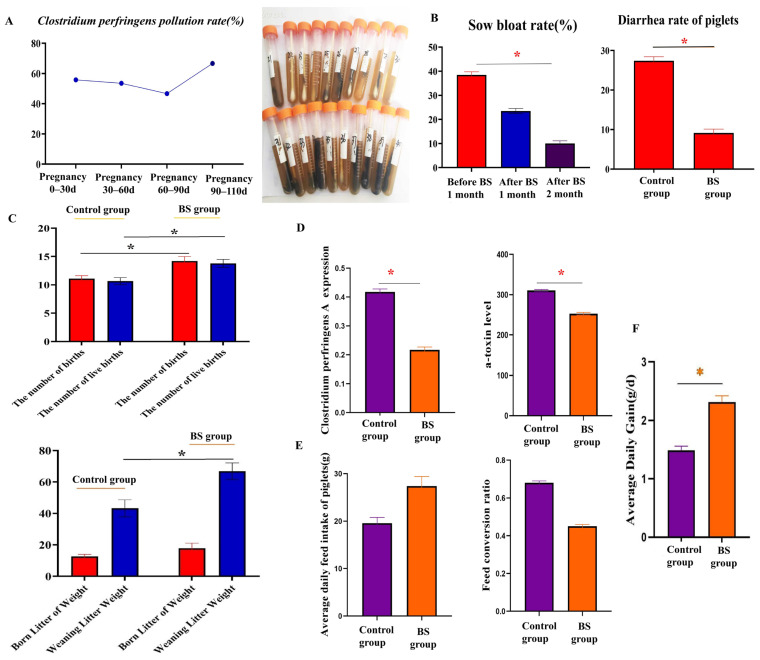
Effects of dietary supplementation with *Bacillus subtilis* PB6 on the reproductive performance of sows. Values are presented as mean ± SD. Statistical analyses were performed using independent samples Student’s *t*-test for all group comparisons, as the study only included two groups (the control group and the *B. subtilis* PB6 group). For multi-time-point data (sow bloat rate in Panel B: before PB6 supplementation, after 1 month of PB6 supplementation, and after 2 months of PB6 supplementation), group comparisons were conducted separately at each time point using independent samples Student’s *t*-test. * *p* < 0.05 significant difference between the experimental and control groups at the same time point. (**A**), *Clostridium perfringens type A* detection rate of sows during the pregnancy period. Black color represents heavy pollution, and brown color represents moderate pollution. (**B**) Sow bloat rate before and after BS feed; diarrhea of piglets in the control and BS group. The number of births, live births, born litter weight, weaning litter weight (**C**), *Clostridium perfringens type A* expression and α-toxin level (**D**), average daily feed intake of piglets and feed conversion ratio (**E**), in the control and BS group. The average daily gain in the control and BS groups (**F**).

The detection rate of *Clostridium perfringens type A* in sows was high at 0–30 d, 30–60 d, 60–90 d and 90–110 d of gestation on this farm (Figure 1A). The detection rate of *Clostridium perfringens* reached up to 50%, and sows were mainly characterized by bloat and an increased mortality rate. We determined that the condition in sows was caused by *Clostridium perfringens type A* infection. Meanwhile, the levels of *Clostridium perfringens type A* and α-toxin in the *B. subtilis* group decreased significantly compared with those in the control group (*p* < 0.05) (Figure 1D).

We supplemented the sow diet with and observed the inhibition of *Clostridium perfringens type A* proliferation. The bloat rate in sows was significantly reduced after feeding *B. subtilis* (*B. subtilis* PB6 group) for 1 and 2 months, and the diarrhea rate in neonatal piglets was significantly reduced following maternal supplementation with *B. subtilis* PB6 (Figure 1B). Both total births and live births were significantly higher in the *B. subtilis* group than in the control group (*p* < 0.05). Both birth litter weight and weaning litter weight were significantly higher in the *B. subtilis* group than in the control group (*p* < 0.05) (Figure 1C). The average daily feed intake of piglets in the *B. subtilis* group was increased compared with that of the control group (*p* < 0.05). The feed conversion ratio was significantly lower in the control group than in the *B. subtilis* group (*p* < 0.05) (Figure 1E). The average daily gain in *B. subtilis* group was significantly increased compared with that in the control group (*p* < 0.05) (Figure 1F).

### 3.2. Effect of PB6 on the Intestinal Microbiological Flora of Sows and Piglets

**Figure 2:**
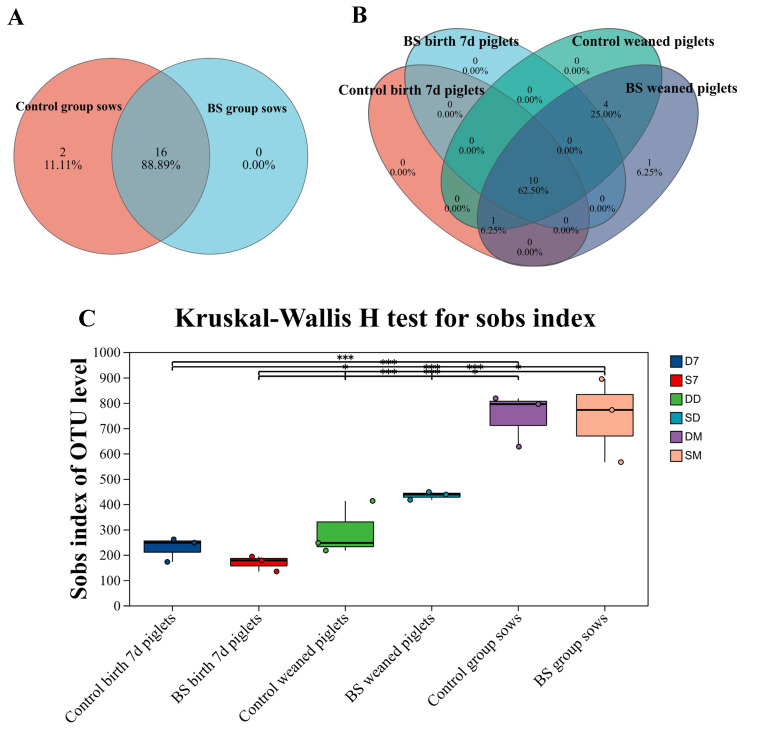

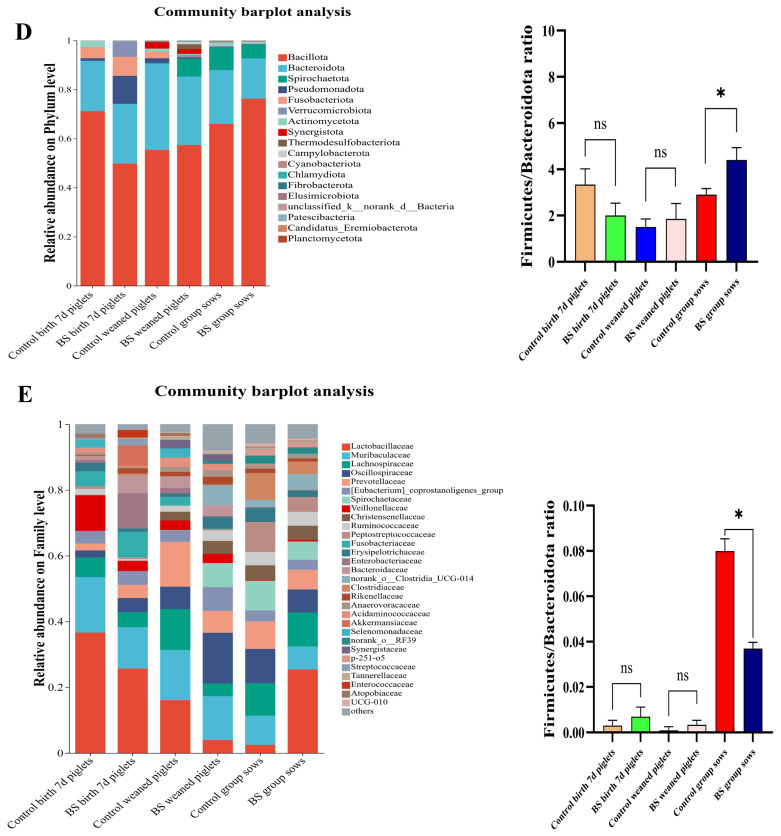

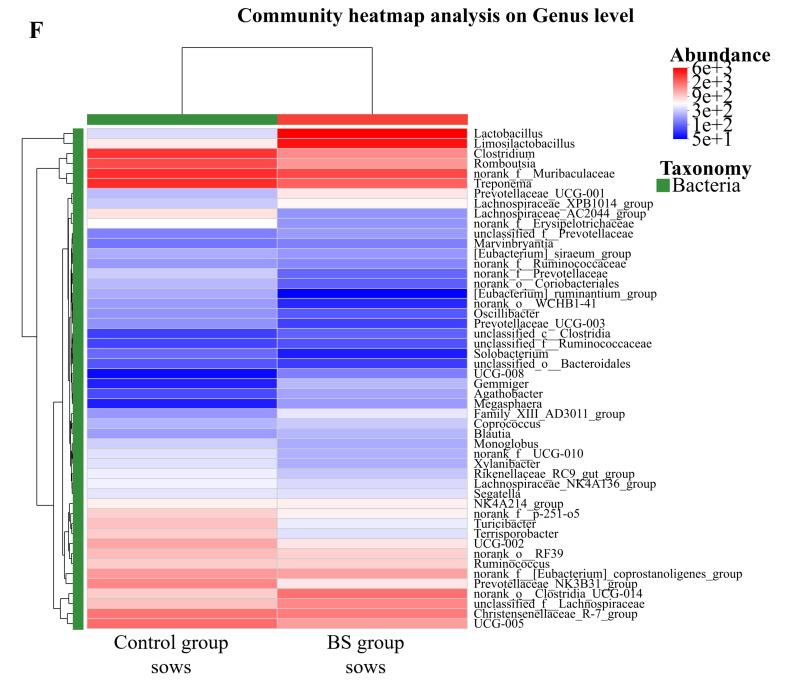

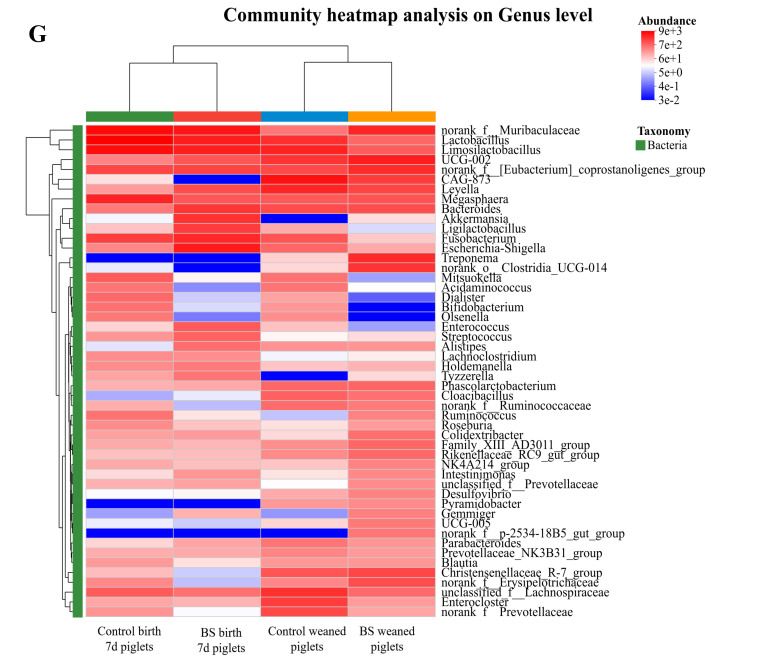

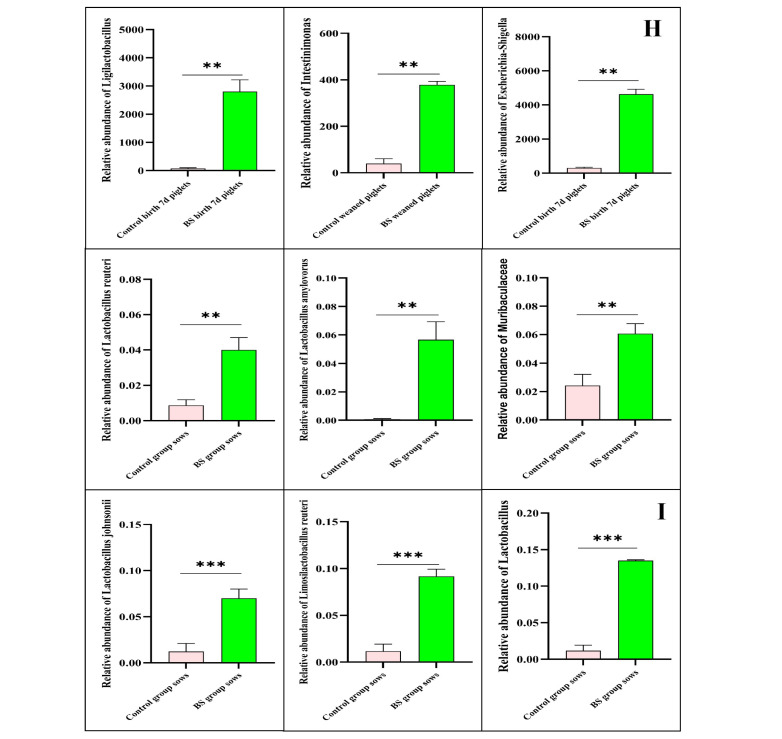
Effect of feeding *Bacillus subtilis* on diversity of the intestinal microbiology in sow and neonatal piglets. Statistical analyses were performed using independent samples Student’s *t*-test for all group comparisons, as the study only included two groups (control group and *B. subtilis* PB6 group). For the same microbiological flora of sows, (**A**) birth 7 d and weaned piglets, and (**B**) between control and BS group. (**C**) Kruskal–Wallis H test for sobs index of control and BS group in sows, birth 7 d, and weaned piglets. Relative abundance on the phylum level is shown in (**D**) and family level. (**E**) In community barplot analysis in sows, birth 7 d and weaned piglets of control and BS group. (**F**) Community heatmap analysis on the genus level of sows in BS and control group. Community heatmap analysis on the genus level of piglet sows. (**F**) and birth 7 d and weaned piglets (**G**) of control and BS group; (**H**) relative abundance of *Lactobacillus*, *Limosilactobacillus reuteri*, *Lactobacillus johnsonii*, *Muribaculaceae*, *Lactobacillus amylovorus*, *Lactobacillus reuteri* in sows of control and BS group. (**I**) Relative abundance of *Escherichia-shigella*, *Ligilactobacillus*, and *Intestinimonas* in birth 7 d and weaned piglets of control and *B. subtilis* group. * *p* < 0.05 significant difference between the experimental and control groups at the same time point. ** *p* < 0.01 significant difference between the experimental and control groups at the same time point. *** *p* < 0.001 significant difference between the experimental and control groups at the same time point.

To further investigate the mechanism by which PB6 reduces the abundance of *Clostridium perfringens type A*, we analyzed the intestinal microbiota of sows and their piglets. Figure 2A reveals that there are 18 core intestinal microbiota between the control and the *B. subtilis* group sows, including *p_Firmicutes*, *p_Bacteroidota*, *p_Spirochaetota* and *p_Actinomycetota* et al. Figure 2B shows the identification of 10 core phyla intestinal microbiota in the 7-day-old piglets and weaned piglets in the control and the *B. subtilis* group, including *p_Firmicutes*, *p_Bacteroidota*, *p_ Fusobacteriota*, *p_ Pseudomonadota*, et al. Furthermore, no significant differences were observed in Sobs indices among groups between *B. subtilis* and control treatments: (a) 7-day-old piglets, (b) weaned piglets, and (c) sows (all *p* > 0.05).

The results of the analysis at the phylum level showed *Bacillota* and *Bacteroidota* are the two phyla with the highest relative abundance in each group. The *Firmicutes*/*Bacteroidota* ratio of sows of the *B. subtilis* group was significantly increased compared to the control group (*p* < 0.05) (Figure 2D). Similarly, at the family level, *Lactobacillaceae* and *Muribaculaceae* are the two families with the highest relative abundance in each group. The relative abundance of Clostridiaceae was significantly decreased in the *B. subtilis* group compared to that of the control group (*p* < 0.05) (Figure 2E). Figure 2F,G shows the genus-level heatmap analyses of gut microbiota for sows and neonatal piglets, respectively. At the genus level of sows, *Lactobacillus*, *Limosilactobacillus reuteri*, *Lactobacillus johnsonii*, *Muribaculaceae*, *Lactobacillus amylovorus*, and *Lactobacillus reuteri* relative abundance of sows in the *B. subtilis* group was significantly increased compared to the control group (*p* < 0.05) (Figure 2H). The relative abundance of *Escherichia-shigella*, *Ligilactobacillus*, and *Intestinimonas* in 7-day-old piglets of the *B. subtilis* group was significantly increased compared to 7-day-old piglets of the control group (*p* < 0.05) (Figure 2I). Collectively, the abundance of *Lactobacillus* and *Muribaculaceae* probiotics in the intestinal lumen of sows was significantly increased, and the relative abundance of *Clostridiaceae* was significantly decreased after feeding PB6 to sows. Meanwhile, the abundance of *Ligilactobacillus* probiotic bacteria in the intestinal lumen of neonatal piglets increased significantly. This suggests that feeding PB6 to sows enhances the relative abundance of probiotic bacteria in both sows and neonatal piglets.

The Shannon index revealed that the intestinal microbial diversity in the *B. subtilis* group was significantly higher than in the control group (*p* < 0.05), and the Chao1 index increased by 12.3%.

Notably, high inter-individual variability is a well-documented characteristic of microbial community studies. In the present study, the microbial data were derived from three independent sows or litters per group, which represents a small sample size for microbiome research. Such limited sample sizes may exacerbate the impact of inherent microbial variability on result robustness, as microbial community composition can be influenced by multiple factors, including individual host individuality and technical variation. Therefore, the microbial community-related findings reported herein should be interpreted with caution, and the detailed limitations of the small sample size are provided in the Section 4.

### 3.3. Effect of PB6 on the Histomorphology of Organs in Sows

*B. Subtilis* strain PB6 promoted the proliferation of intestinal probiotics and inhibited the growth of pathogenic bacteria. Given the critical role of probiotics in modulating intestinal histomorphology, we further analyzed the histological features of sow organs and the structural integrity of the intestinal mucosal immune barrier.

**Figure 3:**
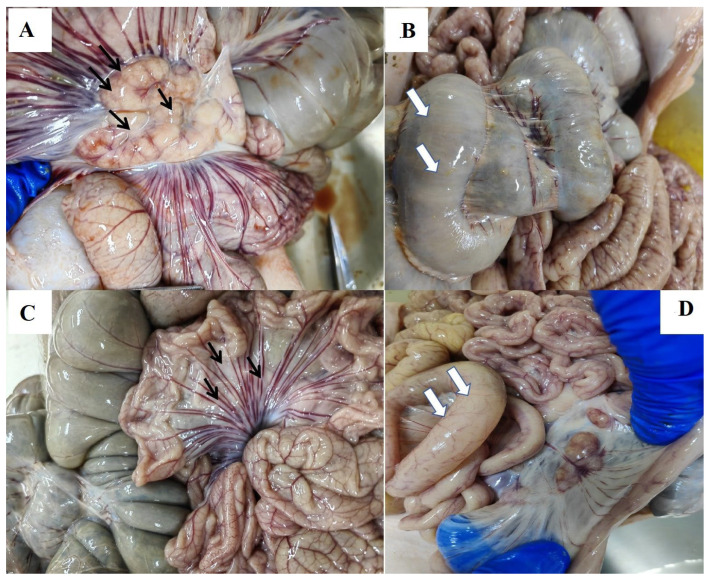

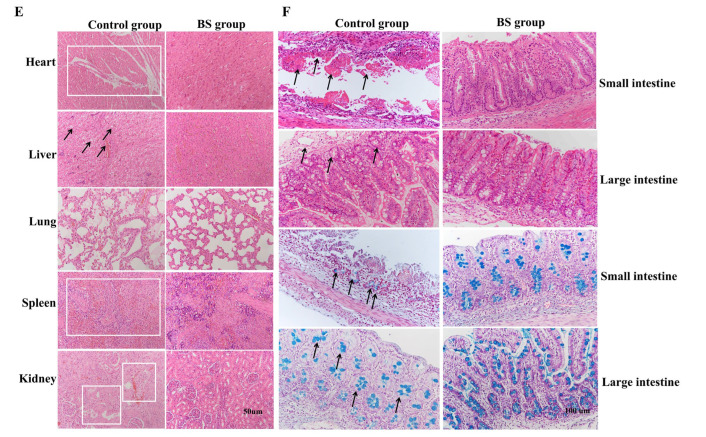
Representative histopathological images of the intestinal tissue of sows and piglets in control and BS groups. Statistical analyses were performed using independent samples Student’s *t*-test for all group comparisons, as the study only included two groups (control group and *B. subtilis* PB6 group). (**A**–**D**) Anatomy of the intestinal tract of the sow. (**E**) The histomorphology of heart, liver, lung, spleen, and kidney after *Clostridium perfringens type A* infection of sows in the control group (feeding normal diets) and BS group (adding *Bacillus subtilis* to diets). (**F**) The histomorphology of intestine after *Clostridium perfringens type A* infection of sow in the control group and BS group, (top two lines were H&E stained, bottom two lines were A&B stained (×200). Scale bar = 100 μm. Arrows indicate mucosal injury or recovery features).

*Clostridium perfringens type A*-infected sows showed severe distension of both the large and small intestines (Figure 3B,D; white arrows), along with extensive congestion, hemorrhage, and edema of the mesentery (Figure 3C; black arrows). Additionally, these sows showed marked enlargement of the mesenteric lymph nodes and intestinal Peyer’s patches (Figure 3A; black arrows).

In the control group, the hearts of sows had myocardial fiber fragmentation with congestion and hemorrhage (white square); extensive hepatocyte necrosis, congestion, and petechiae hemorrhage were observed in the live (black arrow); the lungs of sows had no pathological damage; the indistinct demarcation between red and white pulp of the spleen of sows were indistinguishable (white square); and the number of glomeruli of the kidneys of sows was significantly decreased (white square). However, sows in the *B. subtilis* group exhibited no significant pathological alterations in the liver, heart or kidney (Figure 3E).

The most severe tissue damage in the sows of the control group was the small intestinal. The villi of the small intestines of the sows were completely absent, the folds all disappeared, and the small intestines appeared to have severe congestion; the mucosal epithelial layer of the large intestinal tissues had severe damage, and the crypts of the folds were deepened; There was no significant damage to the histomorphologic structure of the intestinal tract in the *B. subtilis* group. Alcian blue staining revealed a significant decrease in the number of goblet cells in the small and large intestinal mucosa of the control group compared with the *B. subtilis* group (Figure 3F).

### 3.4. Effect of PB6 on the Distribution and Levels of MUC2 and sIgA in Sows and Piglets

**Figure 4:**
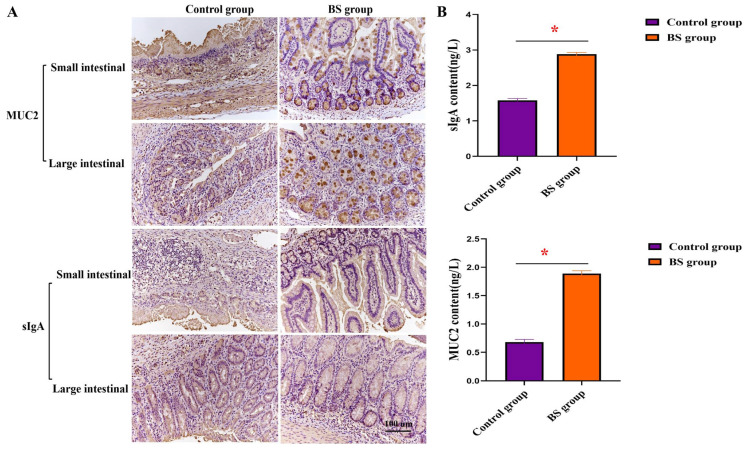
(**A**,**B**) The MUC2 and sIgA (brown color) distribution and level in intestinal were analyzed by immunohistochemistry and ELISA after Clostridium perfringens type A infection in the control group and BS group (×200). * *p* < 0.05 significant difference between the experimental and control groups at the same time point.

We further analyzed the distribution and expression levels of MUC2 and sIgA in the sows’ intestines using IHC. In the *B. subtilis* group, the MUC2 protein is localized within goblet cells and secreted to coat the epithelial cell surface, forming a mucus layer. Secretory immunoglobulin A (sIgA) is primarily expressed on the intestinal mucosa, secreted by plasma cells into the intestinal lumen, and plays a crucial role as the ‘first line of defense’. The volume of the secreted mucus showed a remarkable recovery, correlating with a structurally intact mucosal immune barrier in the *B. subtilis* group. These results indicated that PB6 effectively restored the morphological structure of the intestinal tract and reinforced the integrity of the mucosal immune barrier in infected sows. The expressions of MUC2 and sIgA in the control group were significantly lower than those in the small and large intestine tissues of sows in the *B. subtilis* group (Figure 4).

### 3.5. Effect of PB6 on the Levels of Inflammatory Factors in Sows and Piglets

*Clostridium perfringens type A* induces severe intestinal bloat and inflammatory responses, so we further analyzed the levels of inflammatory immune factors.

The CRP levels were significantly elevated in 7-day-old neonatal and weaned piglets from the *B. subtilis* group compared to the control group (*p* < 0.05). The CRP levels of sows in the control group were significantly increased compared to the *B. subtilis* group (*p* < 0.05); the IgG levels in 7-day-old neonatal piglets and sows from the *B. subtilis* group were significantly increased compared to the control group (*p* < 0.05), with no significant differences between the *B. subtilis* group and control group (*p* > 0.05); SAA levels of sows and weaned piglets in the control group were significantly increased compared to the *B. subtilis* group (*p* < 0.05); cortisol levels of weaned piglets from the control group were significantly increased compared to the *B. subtilis* group (*p* < 0.05); IgM levels of sows, neonatal piglets and weaned piglets in the control group were significantly increased compared to the *B. subtilis* group (*p* < 0.05).

**Figure 5:**
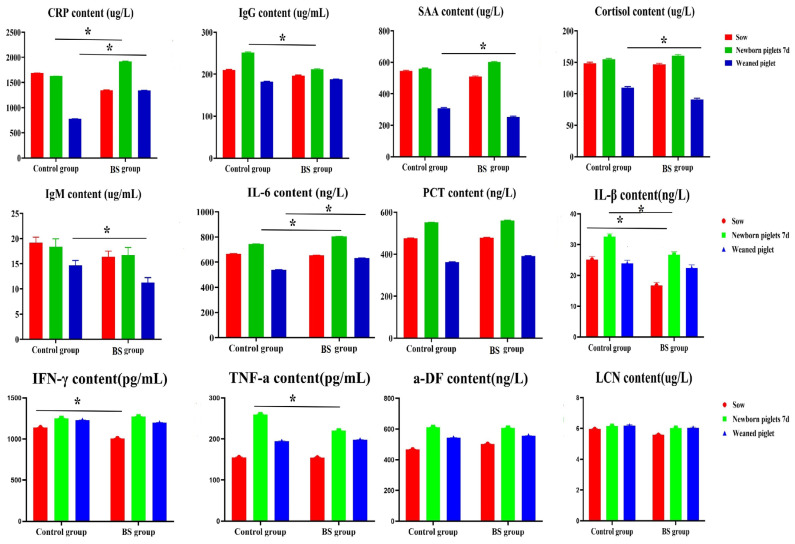
Serum biochemical and immune indices of sows fed diets with or without Bacillus subtilis PB6 supplementation. Statistical analyses were performed using independent samples Student’s *t*-test for all group comparisons, as the study only included two groups (control group and B. subtilis PB6 group). Data are expressed as mean ± SEM. Statistical significance was determined using one-way ANOVA followed by Tukey’s test. * *p* < 0.05 significant difference between the experimental and control groups at the same time point.

IL-6 levels of neonatal piglets and weaned piglets from the *B. subtilis* group were significantly increased compared to the control group (*p* < 0.05); PCT levels of sows, neonatal piglets, and weaned piglets in the control group were not significantly different compared to the *B. subtilis* group (*p* > 0.05). IL-1β levels of sows and neonatal piglets in the control group were significantly increased compared to the *B. subtilis* group (*p* < 0.05); IFN-γ levels of sows in the control group were significantly increased compared to the *B. subtilis* group (*p* < 0.05); TNF-α levels of neonatal piglets in the control group were significantly increased compared to the *B. subtilis* group (*p* < 0.05); there were no significant differences in sows, neonatal piglets and weaned piglets in the control group and *B. subtilis* group (*p* > 0.05) (Figure 5). Collectively, these results demonstrate that PB6 supplementation reduces the inflammatory response in sows and weaned piglets following infection with *Clostridium perfringens type A*.

## 4. Discussion

The primary objective of this study was to evaluate the effects of dietary supplementation with *Bacillus subtilis* PB6 on the reproductive performance of sows infected with *Clostridium perfringens type A*, as well as the growth performance and intestinal health of their offspring.

Our results demonstrated that *B. subtilis* PB6 significantly reduced the relative abundance of *Clostridium perfringens type A* and the level of α-toxin in sows. Thereby, this led to a decreased incidence of intestinal bloating in sows and diarrhea in neonatal piglets. Meanwhile, *B. subtilis* PB6 improved the reproductive performance of sows (including increased total births, live births, birth litter weight and weaned litter weight) and the growth performance of piglets, regulated the intestinal microbiota balance by enhancing the abundance of beneficial bacteria such as *Lactobacillus*, repaired the intestinal tissue structure and mucosal immune barrier, and alleviated the inflammatory response in sows and piglets. These results provide direct scientific evidence supporting *B. subtilis* PB6 as an effective alternative antibiotic for preventing *Clostridium perfringens type A*-induced issues in the swine industry, with important practical application value.

In this study, sows on this farm showed a high incidence of severe intestinal bloating during gestation. This condition significantly impaired reproductive performance and consequently led to a marked increase in diarrhea rate of neonatal piglets. Neonatal piglets often show anorexia, slow growth, low feed utilization, and diarrhea due to maternal poor health [9]. Therefore, our preliminary analysis attributed this sow’s bloating primarily to *Clostridium perfringens type A* infection. CPA is known to induce various conditions, such as food poisoning, gas gangrene, and antibiotic-associated diarrhea through the production of multiple toxins and extracellular enzymes [10,11]. On this experimental farm, natural infection with *Clostridium perfringens A* during gestation was responsible for a reduction in total births and live births, birth litter weight and weaning litter weight. The above tests indicated that dietary supplementation with PB6 significantly reduced the intestinal abundance of *Clostridium perfringens type A* and the levels of released α-toxin in pregnant sows, improved reproductive performance, and decreased the incidence of diarrhea in neonatal piglets.

To investigate whether *B. subtilis* could alleviate intestinal bloat in pregnant sows, the results showed that dietary supplementation with *B. subtilis* PB6 protected neonatal offspring from *Clostridium perfringens type A* diarrhea by enhancing their immune response against *Clostridium perfringens type A* and transferring functional maternal antibodies to their offspring. *B. subtilis* PB6 could inhibit the proliferation and metabolism of *Clostridium perfringens type A* and significantly reduce the levels of α-toxin.

To further explore the mechanism underlying the inhibition of *Clostridium perfringens type A* colonization, we analyzed the diversity of intestinal microbiota in pregnant sows and neonatal piglets. The gut microbiota plays a crucial role in utilizing nutrients, producing volatile fatty acids and vitamins, regulating the immune system, and enhancing resistance against enteric pathogens. Gut microbial diversity and composition can be shaped by feed additives, such as antibiotics and probiotics [12,13,14]. Following supplementation with *B. subtilis* PB6, the sows’ intestinal lumen *Lactobacillus* spp., *Limosilactobacillus reuteri*, *Lactobacillus johnsonii*, *Muribaculaceae* spp., *Lactobacillus amylovorus*, and *Lactobacillus reuteri* relative abundances were significantly increased compared with the control group. These results indicate that *B. subtilis* PB6 can promote a significant increase in the abundance of *Lactobacillus*-related intestinal probiotics [15,16,17]. This regulatory effect on beneficial intestinal flora is consistent with findings in hemorrhagic bowel syndrome (HBS) research, a swine intestinal disease also closely associated with *Clostridium perfringens type A* infection. Studies on HBS have demonstrated that housing stress (e.g., high stocking density) or feed-induced dysbiosis can exacerbate CPA colonization, while supplementation with probiotics (similar to PB6) increases *Lactobacillus* abundance to compete with CPA for intestinal niches, thereby reducing HBS severity.

Numerous studies have demonstrated that PB6 exerts a significant inhibitory effect on CPA. As shown in Reference [18], PB6 may inhibit the expression of CPA α-toxin by secreting surfactants. These lipopeptide substances possess a unique amphiphilic structure that can disrupt the integrity and function. In this study, significant changes were observed in the α-toxin-related indicators of CPA in the PB6 treatment group, suggesting that surfactants may be involved. Subsequently, LC-MS was employed to detect the content of surfactants in PB6 cultures. This analysis further verified the mechanism and elucidated the molecular pathways of the interaction between PB6 and CPA, providing key data support for the role in regulating intestinal microbiota. *Lactobacillus* can synthesize and secrete a class of proteinaceous inhibitory substances through its metabolic processes; these substances exhibit potent inhibitory activity against cognate bacteria and belong to bacteriocins. Each strain of *Lactobacillus* produces a potent bacteriocin that exhibits potent inhibitory activity toward Gram-positive bacteria. Some studies have shown that dietary supplementation with *B. subtilis* PB6 decreased the abundance of *Clostridium perfringens type A*, while increasing the abundance of *Bacteroidetes* [19,20]. Consistent results were observed in this experiment, and the greatest damage induced by *Clostridium perfringens type A* infection was to the morphology and structure of intestinal tissues, disrupting the mucosal immune barrier of the intestine. When *B. subtilis* PB6 reduces the relative abundance of *Clostridium perfringens type A* and the level of α-toxin, intestinal tissue damage is reduced, and the intestinal tissue morphology and structure are restored, so as to effectively prevent the migration of *Clostridium perfringens type A* into the bloodstream.

In addition, *Muribaculaceae* are a family of bacteria within the order *Bacteroidetes*. *Muribaculaceae* have attracted much attention because of their beneficial roles in maintaining host health [21,22,23,24]. The increased abundance of *Muribaculaceae* may regulate intestinal barrier function by producing short-chain fatty acids, such as propionic acid and butyric acid, and its potential as a next-generation probiotic needs to be further validated through in vitro functional experiments [24]. The upregulation of *Muribaculaceae* gene expression levels facilitates the regeneration of mucins and sIgA production in response to intestinal tissue injury. Consequently, this reduces intestinal permeability, prevents the translocation of pathogenic bacteria, and promotes the restoration of the intestinal tissue structure in sows. *B. subtilis* PB6 enhances intestinal immunity by activating immune cells within the gut, modulating the secretion of both pro-inflammatory and anti-inflammatory cytokines, and increasing the production of secretory immunoglobulins [25]. Meanwhile, probiotics can significantly elevate the levels of serum immunoglobulin M (IgM), immunoglobulin G (IgG), and IL-1β in neonatal piglets, enhance the level of secretory immunoglobulin A (sIgA) in intestinal tissues, stimulate the proliferation and differentiation of immune cells, improve immunoglobulin production, and enhance the function of non-specific and specific immunity [26,27,28]. In this study, probiotics supplementation regulated the production of inflammation-related cytokines in the intestinal lumen, reduced the release of pro-inflammatory factors (CRP, IFN-γ, and TNF-α), enhanced the intestinal barrier function, and protected the intestinal mucosa from damage. Furthermore, the probiotics transmitted via milk during lactation can colonize the intestinal lumen of neonatal piglets, thereby helping them to establish a beneficial microbiota colonized by *Lactobacillus*, inhibit pathogenic bacteria, and reduce the piglets’ inflammatory response.

Although the direct transmission pathway of *B. subtilis* PB6 through milk remains unproven herein, the observed clinical improvements in neonatal piglets (e.g., reduced diarrhea rate, increased average daily gain) still provide a valuable documented correlation between maternal dietary supplementation and offspring health. However, given the absence of direct evidence for immune transfer (e.g., detection of PB6, specific antibodies, or functional immune factors in colostrum or milk), discussions regarding the “maternal–offspring axis” must be approached with caution. Such associations should be framed primarily as an observed correlation rather than a definitive mechanistic pathway linking maternal supplementation to neonatal benefits. This caveat aligns with the study’s limitations and ensures the interpretation remains scientifically rigorous while acknowledging the practical relevance of the observed maternal-offspring health associations.

Taken together, our results suggest that dietary supplementation with *B. subtilis* PB6 could improve the reproductive performance of sows, which is attributed to the ability of *B. subtilis* PB6 to promote the propagation of intestinal probiotics, competitively inhibit the proliferation of *Clostridium perfringens type A,* as well as inhibit the α-toxin production. Furthermore, probiotics in the intestinal lumen of sows can restore the damaged intestinal tissue histomorphology and structure, strengthen the integrity of the intestinal mucosal immune barrier, further inhibit the translocation of *Clostridium perfringens type A*, and alleviate the inflammatory response in sows. However, this relatively small sample size may have limited the statistical power of our analyses. Additionally, the robustness of the association between *B. subtilis* PB6 supplementation and reduced sow bloat could be further reinforced with a larger sample. Future studies should expand the sample size of sows (incorporating multi-batch or multi-farm replicates) and extend the follow-up period of reproductive performance to validate the current findings and improve the generalizability of our conclusions.

## 5. Conclusions

The reduction in *Clostridium perfringens type A* abundance has led to a significant decrease in the α-toxin level, attributable to the *Bacillus subtilis* treatment significantly increasing the abundance of intestinal probiotics in sows and decreasing the relative abundance of *Clostridium perfringens type A*. These probiotics can repair intestinal tissue and histomorphology and enhance the expression of MUC2 and sIgA, and thereby strengthen intestinal mucosal immune function. Additionally, *B. subtilis* reduced inflammatory factor levels and inflammatory responses in sows and neonatal piglets. Collectively, these suggest that dietary supplementation with *B. subtilis* PB6 could alleviate bloat in sows and diarrhea in piglets, while improving intestinal barrier function and microbial balance. These findings offer new strategies for managing *Clostridium perfringens type A*-induced diarrhea in pig farms.

## Figures and Tables

**Table 1:** Judgment results of *Clostridium perfringens* infection.

Culture Time (h)	Color Development Phenomenon	Determination of Viable Bacteria Count (CFU/g)	Clinical Diagnosis of *Clostridium perfringens*
≤2	Black appears	>10^7−8^	For severe infection, treatment is recommended
2–4	Black appears	10^6^–10^7^	For moderate infection, treatment is recommended
4–5	Black appears	10^4^–10^6^	Mild infection. It is recommended to take preventive measures
≥5	does not appear or black appears	<10^4^	The Clostridium count in the intestinal tract of the body is normal

**Table 2:** Primer sequences of the genes.

Gene Name	Primer	Sequences	Product Size (bp)
*Clostridium perfringens type A*	FP	CGTAGGCGGATGATTAAGT	169 bp
	RP	CCTCAGCGTCAGTTACAG	
Bacterial 16S RNA gene	FP	CCTACGGGNGGCGCAG	200 bp
	RP	GACTACHVGGGTATCTAATCC	

## Data Availability

No datasets were generated or analysed during the current study.
